# A Population-Based Cross-Sectional Study of the Association between Liver Enzymes and Lipid Levels

**DOI:** 10.1155/2018/1286170

**Published:** 2018-06-03

**Authors:** Subrata Deb, Prasanth Puthanveetil, Prashant Sakharkar

**Affiliations:** ^1^Department of Pharmaceutical Sciences, College of Pharmacy, Larkin University, Miami, FL 33169, USA; ^2^Roosevelt University College of Pharmacy, Schaumburg, IL 60173, USA

## Abstract

**Background:**

To examine the association between low-density lipoprotein (LDL) and high-density lipoprotein (HDL) levels and liver enzyme functions.

**Methods:**

The National Health and Nutrition Examination Survey (NHANES) data from 1999 to 2012 was used to examine the association between liver enzymes and lipid levels amongst adults in the United States.

**Results:**

Sixteen percent adults had ALT > 40 U/L, 11% had AST > 40 U/L, and 96% had ALP > 120 U/L. Age, gender, and race/ethnicity showed significant association with LDL, HDL, and triglycerides levels. LDL greater than borderline high was associated with little over two times higher odds of elevated ALT (OR: 2.33, 95% CI: 2.17, 2.53, *p* ≤ 0.001) and AST (OR: 2.79, 95% CI: 2.55, 3.06, *p* ≤ 0.001). High HDL was associated with 50% higher odds for elevated ALT (OR: 1.51, 95% CI: 1.39, 1.64, *p* ≤ 0.001) and over two-and-half fold elevated AST (OR: 2.77, 95% CI: 2.47, 3.11, *p* ≤ 0.001). LDL-C, HDL-C, and triglycerides were found to be good predictor of elevated ALT, AST, and ALP levels. Similarly, old age and female gender were significant predictor of elevated ALT and AST (*p* ≤ 0.001).

**Conclusions:**

Underlying hepatic pathophysiology from dyslipidemia deserves further exploration due to its potential effects on hepatic drug metabolism/detoxification.

## 1. Introduction

Elevated lipid induced complications are significantly higher in North America and worldwide. Higher lipid levels are known to cause complications without sparing any organs including central nervous system, cardiovascular and endocrine systems, and even hepatic and renal systems [1]. Even though all the organ systems are affected by elevated lipid levels, liver is considered to be the generator and balancer of lipoprotein particles in the body including low-density lipoprotein (LDL), very low-density lipoprotein (VLDL), and high-density lipoprotein (HDL) [1]. An increase in the cholesterol levels considered to be detrimental, specifically LDL and VLDL, has always been a predictor for cardiovascular disease [1].

Approximately 30% of Americans are living with fatty liver disease, an epidemic that is rising in geometric proportions because of the calorie rich diet, lack of physical activity, and diabetes [2]. Genetically and nongenetically induced lipid disorders can lead to accumulation of excess fat bodies in the liver. This excess lipid thus brings about a change in liver anatomy, physiology, metabolism, and even survival of individual hepatocytes ultimately resulting in fibrosis and damage of hepatic tissue [2]. One of the major consequences resulting from such events could be tremendous oxidative stress that is generated because of the excess lipid accumulation in hepatocytes leading to their inflammation and cell death [2].

Even though there are published studies that have investigated the effect of lipids on cardiovascular system [3], there are limited studies which have looked into the impact of lipids on liver function. The goal of the present study was to examine the association between LDL and HDL levels and liver enzyme functions in the population of United States (US). We have utilized National Health and Nutrition Examination Survey (NHANES) of noninstitutional civilian population of the US to accumulate data for this particular study. The prominent aminotransferases such as alanine amino transferases (ALT) and aspartate aminotransferases (AST) are considered as markers of hepatic stress or injury [4]. ALT is predominantly a hepatic residing enzyme but also found in muscle tissues and kidneys. ALT levels tend to increase in population suffering from viral attack on hepatic tissues, metal overloads, and metabolic stress, whereas AST is a rate limiting enzyme involved in transamination reaction which exists in cytosolic and mitochondrial compartments [4]. Like ALT, AST is also found in liver, muscle tissues, and kidneys, with their levels tending to increase in case of high alcohol consumption, chemical, and lipid induced hepatotoxicity [4]. In our study, we have also included the alkaline phosphatase levels (ALP), which have enhanced expression in lipid induced hepatic inflammatory diseases [4]. This work provides us evidence about the connection between high lipids and liver function in the absence of any confounding factors like metabolic syndrome and diabetes.

## 2. Materials and Methods

### 2.1. Study Population

We analyzed the sample of adults 20 years and older who participated in NHANES, an ongoing population-based statistical survey to estimate the health status of the noninstitutionalized civilian US population, based on interview, examination, and laboratory information from representative samples of US households. In-person interviews were conducted in sampled households, and subjects were invited to participate in medical examinations. Participants were selected using a stratified multistage probability design with oversampling of certain age and ethnic groups. We extracted data on individuals with age equal or above 20 years old, who participated in NHANES from 1999 through 2012 into a combined dataset (NHANES 1999–2012) to increase sample size for greater estimator reliability (NHANES Analytic Guidelines) [5]. Of 71,916 total participants who completed a home interview, 68,705 (96%) were screened. We excluded individuals with hepatitis B surface antibody (*n* = 6179), hepatitis B surface antigen, hepatitis D antibody and hepatitis C confirmed antibody combined (*n* = 653), and hepatitis A antibody and hepatitis B core antibody (*n* = 17649). We also excluded participants with missing AST and ALT levels (*n*  = 2532). Participants (*n* = 7249) had missing data on one or more covariates, leaving final adjusted sample of 34,443 participants for analyses.

### 2.2. Covariates

Covariates included age, gender and ethnicity, blood pressure, cardiovascular disease, alcohol use, and use of lipid-lowering drugs. Age, race, and ethnicity were self-reported by the participants.

### 2.3. Study Definitions

Hyperlipidemia was defined in accordance with “National Cholesterol Education Program-Adult Treatment Panel” (NCEP-ATP III) guidelines [6]. Individuals were considered hyperlipidemic, if they had LDL 130 mg/dl or higher; HDL 40 mg/dl or lower in male; and 50 mg/dl or lower in female. Body mass index, a measure of obesity defined as weight in kilograms divided by height in meters squared, was categorized according to clinical guidelines set by the National Institute of Health [7].

### 2.4. Statistical Analysis

The statistical analyses for this study were performed using STATA, version 14 (STATA Corp, College Station, Texas) a statistical software package that takes into account sample weighting and the complex, multistage probability sample design of NHANES [8]. Demographic characteristics were compared by age, gender, race/ethnicity, education, lipid, and glycemic markers using the Chi-square test. Sampling weights were applied to take into account selection probabilities, oversampling, nonresponse, and differences between the sample and the US adolescent male population. We examined the association between lipid levels and ALT or AST. A multivariate logistic regression model was constructed to evaluate the associations of lipid levels with abnormal ALT or AST. There is no universal agreement about the cut-off values for ALT and AST in published literature. In this study, we defined abnormal ALT and AST as values greater than 40 U/L for total sample population. We also used gender based cut-off value of ALT (>40 U/L for male and >31 U/L for female) and AST (>37 U/L for male and >31 U/L for female) to explore if this change in cut-off value produces any difference in associations [9]. These cut-off values were chosen as it represents common institutional reference values and commonly used in the clinical practice. Similar cut-offs have been used in both adolescent and adult epidemiological studies. Regression model was adjusted for age, gender, and ethnicity. For interpretability, we treated lipid values as categories using clinical cut-off values: LDL ≥ 130 mg/dl; HDL ≤ 40 in male and ≤50 in female; triglyceride ≥ 150 mg/dl. Taylor series linearization was used for variance estimation. A *p* value of ≤0.05 or ≤0.001 was considered statistically significant.

## 3. Results

### 3.1. Study Participants

Table 1 presents the demographic and the baseline characteristics of individuals with low, medium, or high LDL, HDL, or triglyceride. A total of 34,443 adults (20 years and above) were included in this study. Sample mean age was 46 ± 0.22 yrs. and 62% individuals were 40 years of age and older, 52% were female, 70% were non-Hispanic White, and 57% had some college or graduate level education.

In overall sample, 27% adults had LDL levels > 130 mg/dl (borderline high) and 19% participants had HDL levels < 40 mg/dl (low). However, based on NCEP-ATP recommendation for female, 40% female had HDL levels less than 50 mg/dl. Twenty-six percent had triglycerides levels greater than 150 mg/dl (borderline high) according to NCEP-ATP III guidelines. Sixteen percent adults had ALT > 40 U/L, 11% had AST > 40 U/L, and 96% had ALP > 120 U/L in overall sample (Table 1).

The mean LDL, HDL, and triglyceride levels were 117.3 mg/dl, 45.8 mg/dl in male and 56.1 mg/dl in female, and 126.9 mg/dl, respectively, all within the normal range, with the exception of HDL being little high. Adults had mean body mass index (BMI) of 28.2 and mean waist circumference of 97.5 cm, well within the normal range with the cut-off values of 30 for BMI, 102 cm for men, and 88 cm for women for waist circumference (Table 2). Mean LDL, HDL, and triglycerides in overall sample among adults who had ALT and AST > 40 U/L was 130 mg/dl and 128.3; 52 mg/dl; 45.4 mg/dl, 50.6 mg/dl; and 157.3 mg/dl and 143.9 mg/dl, respectively, again within the normal limit with the exception of HDL, which was little high (Table 3).

### 3.2. Association of LDL, HDL, and Triglycerides with Abnormal Liver Enzymes

We examined the association of demographic characteristics with LDL, HDL, and triglyceride levels using the Chi-square test. Age, gender, and race/ethnicity showed significant association with LDL, HDL, and triglycerides levels. Post hoc analyses showed that >40 years of age and non-Hispanic white and black ethnicity were significantly associated with high LDL and borderline high triglycerides. Age of sixty years and above was also significantly associated with high triglycerides. Female gender was significantly associated with high HDL (>40 mg/dl) and high triglyceride levels (200–499 mg/dl), respectively. Non-Hispanic black ethnicity was significantly associated with borderline high LDL, Mexican American, and non-Hispanic black ethnicity with low HDL and high triglycerides (200–499 mg/dl). Obesity and presence of cardiovascular disease (CVD) were significantly associated with high HDL, whereas being on lipid-lowering drugs demonstrated significant association with low LDL levels. Blood pressure found significantly associated with triglyceride levels; however, LDL and HDL did not show any significant association with blood pressure status (Supplemental Tables 1(a) and 1(b)). Similarly, drinking five or more alcoholic drinks per day was found to be significantly associated with ALT and AST but showed no association with lipid markers (data not shown).

We also examined the association of LDL, HDL, and triglycerides with liver enzymes. LDL, HDL, and triglycerides were found significantly associated with liver enzymes ALT and AST (Table 4). Post hoc analysis showed low LDL significantly associated with normal ALT and AST levels, whereas, high HDL and triglycerides were associated with elevated ALT and AST. BMI of <30 and blood pressure of 130/90 were found significantly associated with normal ALT and AST in overall sample and in male and female based on gender based cut-off values with the exception of association between blood pressure and ALT in female. Taking lipid-lowering drugs and CVD found associated with normal AST; however, no such association was observed based on gender (Supplemental Table 1(c)).

We calculated the odds ratio of abnormal ALT (ALT > 40 IU/L), AST (AST > 40 IU/L) using cut-off value of borderline high LDL, low HDL, and borderline high triglycerides. LDL greater than borderline high was associated with over two times higher odds of elevated ALT (OR: 2.33, 95% CI: 2.17, 2.53, *p* ≤ 0.001) and AST (OR: 2.79, 95% CI: 2.55, 3.06, *p* ≤ 0.001). High HDL was associated with 50% higher odds for elevated ALT (OR: 1.51, 95% CI: 1.39, 1.64, *p* ≤ 0.001) and over two-and-half fold elevated AST (OR: 2.77, 95% CI: 2.47, 3.11, *p* ≤ 0.001). Triglyceride levels above borderline high were associated with more than two-and-half times higher odds of abnormal ALT (OR: 2.71, 95% CI: 2.51, 2.93, *p* ≤ 0.001) and little over three times higher odds of elevated AST (OR: 3.21, 95% CI: 2.93, 3.52, *p* ≤ 0.001) compared to normal LDL, HDL, and triglyceride levels (Table 5).

We modeled the odds of abnormal ALT and AST using LDL, HDL, triglyceride, and other covariates simultaneously and calculated the odds ratio of abnormal ALT (ALT > 40 U/L), AST (AST > 40 U/L) using logistic regression model (Table 6). After adjustment, high LDL was associated with 7% and 8% higher odds of elevated ALT and AST, respectively, and high HDL was associated with 59% higher odds for abnormal ALT and close to threefold higher odds for abnormal AST (OR: 1.59; 95% CI: 1.01, 1.13, *p* ≤ 0.001 and OR: 2.85; 95% CI: 1.01, 1.16, *p* ≤ 0.001, respectively). Whereas, high triglyceride levels were associated with 29% higher odds of higher ALT and 43% higher odds of elevated AST (OR: 1.29; 95% CI: 1.21, 1.37, *p* ≤ 0.001 and OR: 1.43; 95% CI: 1.32, 1.55, *p* ≤ 0.001, respectively). Age was also significantly associated with lower odds of having elevated ALT (OR: 0.87; 95% CI: 0.85, 0.89, *p* ≤ 0.001) and AST (OR: 0.94; 95% CI: 0.91, 0.97, *p* ≤ 0.001) and female with lower ALT (OR: 0.49; 95% CI: 0.46, 0.53, *p* ≤ 0.001) and AST (OR: 0.75; 95% CI: 0.69, 0.82, *p* ≤ 0.001) suggesting that increase in age and female gender lowers the odds of elevated ALT and AST and seems to be protective.

### 3.3. Association of LDL, HDL, and Triglycerides with Abnormal Liver Enzymes Using Gender Based Cut-Off Values.

Twenty percent male and 11% female had elevated ALT, and 9% male and 10% female had elevated AST according to the gender based cut-off values (data not shown). Mean ALT in male and female was 29.9 U/L and 21.5 U/L, respectively. Similarly, mean AST in male was 27.2 U/L and in female 23.3 U/L (Table 2).

Mean LDL, HDL, and triglycerides among male who had ALT > 40 U/L and AST > 37 was 124.6 mg/dl and 121.4 mg/dl; 43.3 mg/dl and 48.2 mg/dl; and 191.2 mg/dl and 177 9 mg/dl, respectively. In case of female, these values were 120.9 mg/dl and 121.2 mg/dl; 53 mg/dl and 56.7 mg/dl; and 160.9 mg/dl and 154.7 mg/dl, respectively, little higher for HDL and triglycerides. These differences in LDL, HDL, and triglycerides with ALT and AST were significant in both genders with the exception of HDL and AST in female (*p* ≤ 0.001) (Table 3). LDL, HDL, and triglyceride levels were found significantly associated with gender-specific ALT and AST cut-off values. Post hoc analysis showed that very high LDL, HDL, and borderline high triglycerides were significantly associated with elevated ALT in male, whereas low HDL and borderline high triglycerides associated with elevated ALT in female. Similarly, borderline high triglycerides were associated with elevated AST in both genders (*p* ≤ 0.05) (data not shown).

LDL greater than borderline high was associated with 39% higher odds (OR: 1.39; 95% CI: 1.18, 1.6, *p* ≤ 0.001); high HDL was associated with 34% higher odds (OR: 1.34; 95% CI: 1.17, 1.55, *p* ≤ 0.001) of ALT in male. Triglyceride above borderline was associated with 83% higher odds (OR: 1.83; 95% CI: 1.59, 2.09, *p* ≤ 0.001) and 36% higher odds (OR: 1.36; 95% CI: 1.03, 1.81, *p* ≤ 0.05) of ALT in female, respectively (Table 5). On regression analysis, after adjustment, high LDL was associated with 38% higher odds (OR: 1.38; 95% CI: 1.18, 1.62, *p* ≤ 0.001) and triglycerides with 87% higher odds (OR: 1.87; 95% CI: 1.63, 2.14, *p* ≤ 0.001) of ALT. Similarly, high HDL was associated with 38% and 21% of higher odds of elevated ALT (OR: 1.38; 95% CI: 1.20, 1.59, *p* ≤ 0.001) and AST (OR: 1.21; 95% CI: 1.01, 1.47, *p* ≤ 0.001) in male. Race/ethnicity and HDL were significant predictor of elevated ALT and AST and age of elevated AST in both genders. Similarly, LDL and triglyceride levels were significant predictors of ALT in male (*p* ≤ 0.001) (data not shown).

## 4. Discussion

In the present study, we have expanded the data to include population of 34,443 individuals with age ranging from 20 years and above who are free of hepatitis A, B, C, and D. Also, we have included participants from 1999 to 2012, which is a wider time range than any of the previous studies performed using NHANES. This study helps in drawing the link between high lipid profile and its injurious effect on liver function as measured by AST, ALT, and ALP levels. The mean age of the study population was 46 years old with 62% ranging over 40 years old and 57% of the participants included had a college or graduate level education. Participants of age 60 years and above (2.6%) had normal ALT compared to other age groups; however, no single group found significantly different for AST levels. Similarly, less number of female compared to male (6% versus 9.8%) had elevated ALT and (5.2% versus 5.9%) had elevated AST on Chi-square analyses (data not shown). On the other hand, overall increase in age and female gender appears to be protective to liver health as indicated by the results of regression analysis.

The mean lipid profile and BMI in our study show an interesting pattern, because the mean LDL, HDL, and triglycerides, along with waist circumference and BMI, were all in the upper part of the borderline, with HDL mean value being relatively higher which is 50.8 mg/dl predicted to offer protection. Individuals on lipid-lowering drugs or with BMI < 30 are more likely to have normal liver function than the others with elevated lipid levels. Frequent and excessive consumption of alcoholic drinks can lead to increased AST/ALT functions but potentially without any interference with the lipid levels. Typically, a healthy liver in a subject who is involved with a more sedentary work and life style will metabolize and maintain a normal systemic lipid level in the body, by increasing hepatic reverse cholesterol transport and also uptake of VLDL and LDL by increasing the LDL receptor expression and concentration on the hepatic surface [10]. This LDL receptor mediated increased uptake of lipid remnants which try to maintain a normal lipid profile in the body, but at the same time the accumulated remnants get stored in liver which could initiate metabolic alterations and induce fibrosis and cell death of the hepatic tissue, also resulting in a dysfunctional hepatic system [11]. Relentless assault by lipids on liver while it performs regular hepatic functions adds more workload on liver and thereby alters its physiology. One of the most important factors to observe is that the accumulated lipid remnants could promote inflammation of hepatic tissue with generation of free radicals inside [11]. These free radicals could then be playing a major role in inducing fibrosis or cell death of the hepatic tissue. The damaged liver cell can release more transaminases outside and can become more permeable due to thinning from the excess stretch caused by accumulated lipid remnants or due to induction of porous and fibrous hepatic tissue by the lipid metabolites.

Our results suggest that in general there are no gender-specific differences in the systemic LDL and HDL-cholesterol levels; however, the LDL levels were slightly higher in females than males, albeit without any statistical significance. The non-Hispanic whites had a higher limit of both borderline and higher levels of LDL compared to Mexican Americans and Hispanics who had the lowest. In addition, non-Hispanic white had higher HDL levels, which reflect the finding of previous studies, where higher lipid level including HDL-cholesterol was accompanied by an increase in hepatic transaminase expression [12]. Increasing age was associated with enhanced triglyceride levels; gender was significantly associated with elevated triglycerides and ethnicity showing a significant trend in the triglyceride increases. Non-Hispanic white population shows elevated triglyceride levels along with LDL and HDL, further indication of diet pattern of specific ethnicities.

There is a significant rise in hepatic AST and ALT enzymes in proportion to raised LDL, HDL, and triglyceride levels. These findings further strengthen the claims and give a projection about the connection between the etiology and epidemiology of lipid induced hepatic dysfunction. There are substantial scientific evidence that shows how lipids can affect liver function in preclinical and also in clinical setting [13, 14]. The antioxidant capacity of human liver could be compromised with increasing lipid levels, resulting in more inflammation and reactive oxygen species in the body especially liver [11, 13]. This could result in many of the liver functions including reverse cholesterol transport, the innate ability by which liver try to keep the levels of bad cholesterol-LDL in check. Also the reactive oxygen species could hinder hepatic ability to uptake remnants through LDL receptor, scavenger receptors like SR-1, and its own ability to pack the remnant cholesterol to fully fledged lipoprotein bodies which the system can actually utilize. No significant association was observed for different lipid levels (optimal to very high) with gender on Chi-square analysis; however, gender was found to be a good predictor of the hepatic function as determined by high AST and ALT levels in male compared to female on regression analysis.

One of the previous studies by Jiang et al. [12] showed that low LDL and high HDL-cholesterol levels, which is considered beneficial, showed a negative correlation between liver function as measured by elevated levels of transaminase enzyme. Authors in this study excluded subjects who had viral hepatitis and positive HCV status and also those on lipid-lowering medications along with those subjects whose ALT or AST data was not available. As authors stated, there were some major confounding factors which could have altered both lipid profiles and liver function as seen from the clinical and laboratory data of those participated individuals [12]. Some of the other studies that have used the NHANES data also showed a correlation between hepatic enzyme levels and metabolic dysfunction. A study by Kim et al. [15] showed an interesting phenomenon that even a small rise in AST and ALT showed characteristic features of metabolic syndrome like effects. Samadi et al. [16] in their study showed that there is a strong correlation between waist circumference, a mark of obesity to liver enzymes, AST, ALT, ALP, and gamma glutamyl transpeptidase (GGT) and also noted a significant correlation that this connection between hepatic enzymes and waist circumference only existed in Hispanic females and not in non-Hispanic black males and females.

Another interesting study by Tsai et al. [17] showed a good correlation between obesity, alcohol consumption, and liver enzyme functions using NHANES data for a 3-year period (2005–08) from more than 8300 adults with ages > 20 years as sample size found that obese females who are excess drinkers tend to have an increased AST, ALT, and GGT levels compared to nonobese and nondrinker females. Recently, Unalp-Arida and Ruhl [18] have reported NHANES data (1988–1994) with twenty-three years of mortality follow-up and suggested a strong link between hepatic disease-induced mortality and enzyme function. A patient population of 14,527 who were not tested positive for viral hepatitis B and C were chosen; also this population was not independently associated with mortalities due to other complications like cancer, diabetes, and cardiovascular disease. Individuals having hepatic steatosis with elevated ALT, AST, and GGT enzymes tend to show an elevated level of mortality due to liver disease. One important point to emphasize in our study is that gender is the only independent determinant of liver function independent of the age, ethnic background, or lipid levels in these individuals. It is important to look into the details of protective effect of female hormones, which are active during the premenopausal state and could play a role in both regulating lipid levels and exhibiting hepatoprotective function. In women who are in the postmenopausal state, this hepatic protective function of estrogen could be lost which could result in elevated AST and ALT levels.

Liver plays a vital role in the biotransformation, detoxification, and excretion of diverse lipophilic agents including medications, dietary substances, and environmental toxicants. In addition to detoxification, the drug metabolizing enzymes also play an important role in converting the prodrugs to their active form. Cytochrome P450 (CYP) and uridine diphosphate (UDP) glucuronosyltransferases (UGT) superfamily metabolizing enzymes primarily execute their functions in liver [19]. Expression and activities of hepatic CYP and UGT enzymes are closely regulated by several physiological and external factors including circulating and tissue lipid levels [20]. Thus, individuals with higher lipid levels are likely to be susceptible to altered metabolism profile. A fatty liver with inefficient functioning could lead to decrease in its ability to detoxify drugs and toxins or activate the prodrugs. This will eventually aggravate systemic oxidative stress and result in vital organ damage and elevating the morbidity and mortality in subjects suffering from nonalcoholic fatty liver disease (NAFLD) [21]. A detailed mechanistic understanding of hyperlipidemia on CYP and UGT levels would facilitate our understanding of therapeutic outcome following hyperlipidemia-related NAFLD.

Our study has some limitations. First, we used ALT and AST as a surrogate marker, which is an indirect assessment of liver dysfunction or liver diseases. Second, the proportions of people with LDL, triglycerides, high ALT, and AST were comparatively small. Therefore, despite the large dataset, the power can be limited in these categories, especially when the sample size is reduced for sensitivity analysis. Third, only a single measurement of ALT and AST levels was available for each individual in the NHANES data. Though a small number of participants had CVD, were on lipid-lowering drugs, and reported drinking more than 5 alcoholic drinks per day, perhaps they may have influenced our results. Similarly, missing data and presence of reported/unreported confounding factors such as smoking, cancer/malignancy, diabetes, and presence of liver conditions among participants are the other factors that may have contributed to our results limiting its generalizability.

## 5. Conclusions

Both high LDL and HDL were associated with significantly higher odds of elevated liver enzymes in the general US adult population. Age, gender, and race/ethnicity have significant association with LDL, HDL, and triglycerides levels. Our findings raise concerns about potentially unrecognized hepatic dysfunction among people with high LDL or HDL and the underlying hepatic pathophysiology can impact hepatic detoxification of drugs or environmental toxicants.

## Figures and Tables

**Table 1:** Demographic characteristics and lipid and liver enzyme laboratory data.

	*N* (%)
*Age (yrs.)*	
20–29	6256 (19)
30–39	5907 (19)
40–49	5754 (21)
50–59	4817 (17)
≥60	11709 (24)

*Gender*	
Male	16538 (48)
Female	17905 (52)

*Ethnicity*	
Mexican American	6518 (7.9)
Other Hispanics	2473 (5.3)
Non-Hispanic White	16275 (70)
Non-Hispanic black	7107 (11)
Other	2070 (5.8)

*Education*	
<High school	8530 (18.5)
High school	7050 (24)
Some college degree	8264 (30)
Graduate degree and above	6163 (27)

Participant with BMI ≥ 30	7504 (34.8)
Participants with Hypertension	2883 (12.0)
Told by Doctor of having high cholesterol	3417 (10.6)
On lipid lowering drugs	1123 (7.8)
Five or more alcohol drinks/day	2065 (15.0)

*LDL (mg/dl)*	
Optimal (<100)	8514 (41.6)
Near optimal/above optimal (100–129)	6338 (30.9)
Borderline high (130–159)	3725 (18.2)
High (160–189)	1373 (6.7)
Very high (≥190)	502 (2.3)

*HDL (mg/dl)*	
<40	6164 (19)
≥40	28279 (81)

*Triglyceride (mg/dl)*	
Normal (<150)	15846 (74.1)
Borderline high (150–199)	2620 (12.3)
High (200–499)	2693 (12.6)
Very high (≥500)	214 (1.0)

*Total Cholesterol (mg/dl)*	
Desirable (<200)	17220 (51)
Borderline high (200–239)	9878 (29)
High (≥240)	7345 (20)

*ALT (U/L)*	
≤40	28828 (84)
>40	5615 (16)

*AST (U/L)*	
≤40	30200 (89)
>40	4243 (11)

*ALP (U/L)*	
≤120	1047 (3.6)
>120	33396 (96)

**Table 2:** Mean values of lipid biomarkers and liver enzymes among participants.

	Mean (95% CI)
LDL (mg/dl)	117.3 (116.5, 118.2)
HDL (mg/dl)	50.8 (50.1, 51.5)
HDL (mg/dl) Male	45.8 (45.1, 46.5)
HDL (mg/dl) Female	56.1 (55.1, 57.1)
Triglyceride (mg/dl)	126.9 (124.9, 128.9)
Total cholesterol (mg/dl)	202.1 (199.6, 204.6)
ALT (U/L)	25.4 (25.0, 25.8)
ALT (U/L) Male	29.9 (29.5, 30.4)
ALT (U/L) Female	21.5 (21.0, 21.9)
AST (U/L)	25.2 (24.8, 25.5)
AST (U/L) Male	27.2 (26.9, 27.5)
AST (U/L) Female	23.3 (23.0, 23.5)
ALP (U/L)	68.9 (68.2, 69.7)
Waist circumference (cm)	97.5 (97.1, 98.0)
BMI	28.2 (28.0, 28.3)
SBP (mmHg)	119.5 (119.0, 119.9)
DBP (mmHg)	71.2 (70.9, 71.6)

ALT: alanine transaminase; AST: aspartate aminotransferase; ALP: alkaline phosphatase; SBP: systolic blood pressure; and DBP: diastolic blood pressure.

**Table 3:** Mean lipid biomarkers and liver enzymes.

	ALT (U/L) [Mean (SE)]			AST (U/L) [Mean (SE)]		
	<40	>40	*p*-value	<40	>40	*p*-value

Overall Sample						
LDL (mg/ml)	122.3 (0.9)	130.8 (3.1)	**≤0.001** ^**∗∗∗**^	123.0 (0.9)	128.3 (3.9)	**≤0.001** ^**∗∗∗**^
HDL (mg/dl)	52.0 (0.4)	45.4 (1.0)	**≤0.001** ^**∗∗∗**^	51.4 (0.4)	50.6 (2.0)	**≤0.001** ^**∗∗∗**^
Triglyceride (mg/dl)	129.5 (1.8)	157.3 (6.8)	**≤0.001** ^**∗∗∗**^	131.8 (1.8)	143.9 (9.7)	0.499

	<40	>40	*p*-value	<37	>37	*p*-value

Male						
LDL (mg/ml)	117.3 (0.6)	124.6 (1.5)	**≤0.001** ^**∗∗∗**^	118.1 (0.5)	121.4 (1.8)	**≤0.05** ^**∗**^
HDL (mg/dl)	46.3 (0.4)	43.3 (0.7)	**≤0.001** ^**∗∗∗**^	45.6 (0.4)	48.2 (0.9)	**≤0.05** ^**∗**^
Triglyceride (mg/dl)	147.2 (2.6)	191.2 (6.9)	**≤0.001** ^**∗∗∗**^	151.8 (2.5)	177.9 (9.9)	**≤0.001** ^**∗∗∗**^

	<31	>31	*p*-value	<31	>31	*p*-value

Female						
LDL (mg/ml)	116.7 (0.5)	120.9 (1.9)	**≤0.001** ^**∗∗∗**^	116.7 (0.5)	121.2 (1.9)	**≤0.05** ^**∗**^
HDL (mg/dl)	56.5 (0.5)	53.0 (1.2)	**≤0.001** ^**∗∗∗**^	56.0 (0.4)	56.7 (1.6)	0.630
Triglyceride (mg/dl)	123.9 (1.3)	160.9 (7.5)	**≤0.001** ^**∗∗∗**^	125.1 (1.3)	154.7 (8.5)	**≤0.001** ^**∗∗∗**^

SE = standard error (SE); significant at ^*∗*^*p* ≤ 0.05 and ^*∗∗∗*^*p* ≤ 0.001.

**Table 4:** Association of lipid markers and liver enzymes (overall sample).

	ALT (U/L) (*N*, %)		*p*-value	AST (U/L) (*N*, %)		*p*-value
	<40	>40		<40	>40	
LDL (mg/dl)						
<100	4471 (33.3)	420 (27.6)	**≤0.001** ^**∗∗∗**^	4595 (32.7)	296 (33.6)^a^	**≤0.05** ^**∗**^
100–129	4454 (33.2)	483 (31.8)		4654 (33.1)	283 (32.2)	
130–159	2969 (22.1)	378 (24.9)		3167 (22.5)	180 (20.5)	
160–189	1125 (8.3)	162 (10.7)		1209 (8.6)	78 (8.9)	
≥190	401 (2.9)	75 (4.9)^a^		433 (3.0)	43 (4.9)^a^	

HDL (mg/dl)						
<40	5417 (17)	747 (2.3)	**≤0.001** ^**∗∗∗**^	5827 (18)	337 (0.94)	**≤0.001** ^**∗∗∗**^
≥40	23411 (68)	4868 (14)		24373 (71)	3906 (10)	

Triglyceride (mg/dl)						
<150	9834 (70.0)	901 (54.9)	**≤0.001** ^**∗∗∗**^	10156 (68.9)	579 (61.2)	**≤0.001** ^**∗∗∗**^
150–199	1976 (14.1)	290 (17.6)		2137 (14.5)	129 (13.6)	
200–499	2074 (14.8)	392 (23.9)^a^		2261 (15.4)	205 (21.6)^a^	
≥500	146 (1.0)	57 (3.5)^a^		170 (1.2)	33 (3.5)^a^	

Significant at ^*∗*^*p* ≤ 0.05 and ^*∗∗∗*^*p* ≤ 0.001. ^a^Differed significantly from others on post hoc analyses.

**Table 5:** Odds of elevated liver enzymes with lipid biomarkers.

	ALT		AST	
	OR (95% CI)	*p* value	OR (95% CI)	*p* value
LDL (mg/dL)	2.33 (2.17–2.53)	**≤0.001** ^**∗∗∗**^	2.79 (2.55–3.06)	**≤0.001** ^**∗∗∗**^
HDL (mg/dL)	1.51 (1.39–1.64)	**≤0.001** ^**∗∗∗**^	2.77 (2.47–3.11)	**≤0.001** ^**∗∗∗**^
Triglyceride (mg/dL)	2.71 (2.51–2.93)	**≤0.001** ^**∗∗∗**^	3.21 (2.93–3.52)	**≤0.001** ^**∗∗∗**^

Male				
LDL (mg/dL)	1.39 (1.18–1.63)	**≤0.001** ^**∗∗∗**^	1.07 (0.865–1.31)	0.551
HDL (mg/dL)	1.34 (1.17–1.55)	**≤0.001** ^**∗∗∗**^	1.19 (0.99–1.44)	0.055
Triglyceride (mg/dL)	1.83 (1.59–2.09)	**≤0.001** ^**∗∗∗**^	1.12 (0.93–1.33)	0.209

Female				
LDL (mg/dL)	0.97 (0.88–1.06)	0.480	0.98 (0.87–1.11)	0.790
HDL (mg/dL)	0.64 (0.49–0.83)	**≤0.001** ^**∗∗∗**^	0.86 (0.61–1.20)	0.360
Triglyceride (mg/dL)	1.36 (1.03–1.81)	**≤0.05** ^**∗**^	0.96 (0.69–1.34)	0.080

OR = odds ratio; CI = confidence interval; significant at ^*∗*^*p* < 0.05 and ^*∗∗∗*^*p* < 0.001.

**Table 6:** Predictors of AST and ALT level on regression (adjusted model).

	ALT		AST	
	OR (95% CI)	*p* value	OR (95% CI)	*p* value
Age	0.87 (0.85, 0.89)	**≤0.001** ^**∗∗∗**^	0.94 (0.91, 0.97)	**≤0.001** ^**∗∗∗**^
Gender	0.49 (0.46, 0.53)	**≤0.001** ^**∗∗∗**^	0.75 (0.69, 0.82)	**≤0.001** ^**∗∗∗**^
Ethnicity	0.93 (0.89, 0.97)	**≤0.001** ^**∗∗∗**^	1.03 (0.98, 1.08)	0.134
LDL (mg/dl)	1.07 (1.01, 1.13)	**≤0.001** ^**∗∗∗**^	1.08 (1.01, 1.16)	0.057
HDL (mg/dl)	1.59 (1.42, 1.78)	**≤0.001** ^**∗∗∗**^	2.85 (2.38, 3.42)	**≤0.001** ^**∗∗∗**^
Triglyceride (mg/dl)	1.29 (1.21, 1.37)	**≤0.001** ^**∗∗∗**^	1.43 (1.32–1.55)	**≤0.001** ^**∗∗∗**^

OR = odds ratio; CI = confidence interval; significant at ^*∗*^*p* < 0.05 and ^*∗∗∗*^*p* < 0.001.

## Abbreviations

LDL: Low-density lipoprotein
HDL: High-density lipoprotein
NHANES: National Health and Nutrition Examination Survey
CDC: Center for Disease Control and Prevention
ALT: Alanine amino transferases
AST: Aspartate aminotransferases
ALP: Alkaline phosphatase levels
BMI: Body mass index
VLDL: Very low-density lipoprotein
GGT: Gamma glutamyl transpeptidase
CYP: Cytochrome P450
UGT: Uridine diphosphate (UDP) glucuronosyltransferases
NAFLD: Nonalcoholic fatty liver disease.

## Supplementary Materials

Supplementary Materials The Supplemental Tables 1(a) and 1(b) illustrate the association of demographic characteristics such as age, gender, and race/ethnicity with different lipid biomarkers such as LDL, HDL, and triglyceride ranges. The Supplemental Table 1(c) highlights the association of demographic characteristics with ALT and AST.
