# Supplementary material for: A Population-Based Cross-Sectional Study of the Association between Liver Enzymes and Lipid Levels
Source: Int J Hepatol. 2018 Jun 3;2018:1286170. doi: 10.1155/2018/1286170 (PMC6008685; doi:10.1155/2018/1286170)
Supplement: Supplementary Materials — The Supplemental Tables 1(a) and 1(b) illustrate the association of demographic characteristics such as age, gender, and race/ethnicity with different lipid biomarkers such as LDL, HDL, and triglyceride ranges. The Supplemental Table 1(c) highlights the association of demographic characteristics with ALT and AST. [file 1286170.f1.zip › 1286170.f1/supplimentary - table 1a may 6, 2018_IJH_2308486.doc]

***Supplemental Tables***

|  | **LDL (mg/dl)** | | | | | | **HDL (mg/dl)** | | |
| --- | --- | --- | --- | --- | --- | --- | --- | --- | --- |
|  | **<100** | **100-129** | **130-159** | **160-189** | **≥190** | **p- value** | **<40** | **≥40** | **p-value** |
| **Age (yrs.)** | **(N, %)** | **(N, %)** | **(N, %)** | **(N, %)** | **(N, %)** |  | **(N, %)** | **(N, %)** |  |
| 20-29 | 1235 (25.2) | 832 (16.7) | 419 (12.5) ^a^ | 128 (9.9) ^a^ | 46 (9.6) ^a^ | ≤0.001*** | 1628 (15) | 372 (3.9) | 0.001*** |
| 30-39 | 835 (17.0) | 875 (17.7) | 559 (16.7) | 221 (17.7) | 80 (16.8) |  | 1506 (15) | 399 (4.3) ^a^ |  |
| 40-49 | 619 (12.7) | 889 (18.0) | 669 (19.9) ^a^ | 256 (19.9) ^a^ | 74 (15.6) |  | 1535 (17) | 395 (4.5) |  |
| 50-59 | 481 (9.8) | 694 (14.0) | 568 (16.9) ^a^ | 241 (18.7) ^a^ | 99 (20.8) ^a^ |  | 1276 (14) | 296 2.8) ^a^ |  |
| ≥60 | 1721 (35.2) | 1647 (33.4) | 1132 (33.8) | 441 (34.3) | 177 (37.2) |  | 3212 (20) | 634 (3.5) |  |
| **Gender** |  |  |  |  |  |  |  |  |  |
| Male | 4233 (49.7) | 3050 (48.1) | 1834 (49.2) | 679 (49.5) | 215 (42.8) | ≤0.05* | 1282 (16) ^a^ | 2651 (32) | ≤0.001*** |
| Female | 4281 (50.3) | 3288 (51.9) | 1891(50.8) | 694 (50.5) | 287 (57.2) ^a^ |  | 538 (6.5) | 3856 (45) ^a^ |  |
| **Race/Ethnicity** |  |  |  |  |  |  |  |  |  |
| Mexican American | 1738 (20.4) | 1999 (18.9) | 687 (18.5) | 247 (18.0) | 69 (13.7) ^a^ | ≤0.001*** | 1614 (6.6) | 428 (1.9) | ≤0.012* |
| Other Hispanic | 625 (7.4) | 484 (7.6) | 323 (8.7) | 116 (8.5) | 33 (6.7) |  | 744 (4.5) | 189 (1.2) |  |
| Non-Hispanic White | 3333 (39.2) | 2750 (43.3) | 1661 (44.6) ^a^ | 611 (44.6) | 246 (49.1) ^a^ |  | 4294 (52) | 1002 (12) |  |
| Non-Hispanic Black | 2099 (24.7) | 1409 (22.3) | 774 (20.8) | 296 (21.6) | 99 (19.8) ^a^ |  | 1890 (11) | 331 (2.2) |  |
| Other | 712 (8.4) | 489 (7.7) | 276 (7.4) | 101 (7.4) | 54 (10.8) |  | 602 (6.2) | 143 (1.6) |  |
| **Obesity** |  |  |  |  |  |  |  |  |  |
| Normal | 2128 (10) | 1802 (9.3) | 1215 (6.1) | 428 (2.1) | 7428 (37) | 0.183 | 3914 (58) ^a^ | 560 (7.6) | ≤0.001*** |
| Obese | 1095 (5.1) | 1066 (5) | 702 (3.3) | 286 (1.3) | 4355 (20) |  | 1959 (26) | 599 (8.5) ^a^ |  |
| **Hypertension** |  |  |  |  |  |  |  |  |  |
| Normal | 1002 (10) | 1177 (12) | 913 (9.4) | 624 (3.8) | 9,750 (51) | 0.101 | 4551 (71) | 1020 (17) | 0.073 |
| High BP | 141 (1.3) | 212 (1.8) | 170 (1.3) | 84 (0.6) | 1031 (8.5) |  | 732 (9) | 188 (2.7) |  |
| **LLD** |  |  | 54 (0.9)  954 (17) |  |  |  |  |  |  |
| LLD | 184 (2.9) ^a^ | 126 (2.3) |  | 10 (0.2) | 42 (0.8) | 0.005* | 251 (5.8) | 52 (1.2) | 0.640 |
| Non LLD drugs | 1667 (29) | 1513 (29) |  | 371 (7) | 622 (11) |  | 3123 (79) | 597 (14) |  |
| **CVD** |  |  |  |  |  |  |  |  |  |
| CVD | 43 (0.3) | 46 (0.3) | 34 (0.3) | 19 (0.09) | 281 (1.8) | 0.495 | 139 (1.7) | 58 (0.6) | 0.031* |
| Other than CVD | 1169 (11) | 1420 (14) | 1098 (10) | 449 (4.2) | 6043 (58) |  | 5474 (79) | 1216 (19) |  |

**Table 1a. Association of demographic characteristics with LDL/HDL lipid biomarkers (Overall sample)**

*Significant at ≤0.05 and ***Significant at ≤0.001; LLD=Lipid lowering drugs; CVD=Cardiovascular disease; BP=Blood pressure, High (≥140/90 mmHg);

^a^=differed significantly with others on post-hoc analyses
