# Supplementary material for: A Population-Based Cross-Sectional Study of the Association between Liver Enzymes and Lipid Levels
Source: Int J Hepatol. 2018 Jun 3;2018:1286170. doi: 10.1155/2018/1286170 (PMC6008685; doi:10.1155/2018/1286170)
Supplement: Supplementary Materials — The Supplemental Tables 1(a) and 1(b) illustrate the association of demographic characteristics such as age, gender, and race/ethnicity with different lipid biomarkers such as LDL, HDL, and triglyceride ranges. The Supplemental Table 1(c) highlights the association of demographic characteristics with ALT and AST. [file 1286170.f1.zip › 1286170.f1/supplimentary - table 1b may 6, 2018_IJH_2308487.doc]

**Table 1b. Association of demographic characteristics with triglyceride lipid biomarker**

|  | **Triglyceride (mg/dl)** | | | | |
| --- | --- | --- | --- | --- | --- |
|  | **<150** | **150-199** | **200-499** | **≥500** | **p-value** |
| **Age (yrs.)** | **(N, %)** | **(N, %)** | **(N, %)** | **(N, %)** |  |
| 20-29 | 2101 (19.6) | 302 (13.3) | 346 (14.3) ^a^ | 22 (10.8) | ≤0.001*** |
| 30-39 | 1914 (17.8) | 343 (15.1) | 389 (15.8) | 29 (14.3) |  |
| 40-49 | 1801 (16.8) | 384 (16.9) ^a^ | 404 (16.4) | 53 (26.1) ^a^ |  |
| 50-59 | 1424 (13.8) | 330 (14.6) ^a^ | 390 (15.8) | 54 (26.6) ^a^ |  |
| ≥60 | 3495 (32.6) | 907 (40.0) | 937 (38.0) ^a^ | 45 (22.2) ^a^ |  |
| **Gender** |  |  |  |  |  |
| Male | 7635 (48.2) ^a^ | 1304 (49.8) | 1437 (53.4) | 54 (25.2) | ≤0.001*** |
| Female | 8211 (51.8) | 1316 (50.2) ^a^ | 1256 (46.6) ^a^ | 160 (74.7) ^a^ |  |
| **Race/Ethnicity** |  |  |  |  |  |
| Mexican American | 2894 (18.3) | 536 (20.5) ^a^ | 620 (23.1) ^a^ | 51 (23.9) | ≤0.001*** |
| Other Hispanic | 1174 (7.4) | 229 (8.8) ^a^ | 220 (8.2) | 22 (10.3) |  |
| Non-Hispanic White | 6318 (39.9) | 1263 (48.2) | 1321 (49.1) ^a^ | 100 (46.9) |  |
| Non-Hispanic Black | 4152 (26.2) ^a^ | 377 (14.4) | 324 (12.0) ^a^ | 26 (12.2) ^a^ |  |
| Other | 1290 (8.2) | 213 (8.1) | 203 (7.6) | 14 (6.6) |  |
| **Obesity** |  |  |  |  |  |
| Normal | 4523 (23) ^a^ | 708 (3.4) | 652 (2.9) | 7118 (36) | ≤0.001*** |
| Obese | 2098 (9.6) | 613 (3) | 633 (3.1) | 7504 (35) ^a^ |  |
| **Hypertension** |  |  |  |  |  |
| Normal | 5553 (30) | 1014 (5.1) | 1022 (4.6) | 9044 (48) | ≤0.001*** |
| High BP | 795 (3.2) | 195 (0.9) | 204 (0.9) | 1639 (7.1) |  |
| **LLD** |  |  |  |  |  |
| LLD | 4058 (29) | 965 (6.6) | 960 (6.6) | 7314 (51) | 0.515 |
| Non LLD | 332 (2.2) | 78 (0.5) | 88 (0.6) | 625 (3.9) |  |
| **CVD** |  |  |  |  |  |
| CVD | 6575 (32) | 1256 (5.9) | 1260 (5.3) | 11299 (54) | 0.058 |
| Other than CVD | 191 (0.7) | 50 (0.18) | 49 (0.18) | 456 (1.6) |  |

*Significant at ≤0.05 and **≤0.001; LLD=Lipid lowering drugs; CVD=Cardiovascular disease;

BP=Blood pressure, High (≥140/90 mmHg); ^a^=differed significantly with others on post-hoc

analyses.
