# Supplementary material for: A Population-Based Cross-Sectional Study of the Association between Liver Enzymes and Lipid Levels
Source: Int J Hepatol. 2018 Jun 3;2018:1286170. doi: 10.1155/2018/1286170 (PMC6008685; doi:10.1155/2018/1286170)
Supplement: Supplementary Materials — The Supplemental Tables 1(a) and 1(b) illustrate the association of demographic characteristics such as age, gender, and race/ethnicity with different lipid biomarkers such as LDL, HDL, and triglyceride ranges. The Supplemental Table 1(c) highlights the association of demographic characteristics with ALT and AST. [file 1286170.f1.zip › 1286170.f1/supplimentary -table 1c may 6, 2018_IJH_2308488.doc]

|  | **ALT (U/L)** | |  | **AST (U/L)** | |  |
| --- | --- | --- | --- | --- | --- | --- |
|  | **<40** | **>40** | **p-value** | **<40** | **>40** | **p-value** |
| **Overall** |  |  |  |  |  |  |
| **BMI (kg/m^2^)** | **(N, %)** | **(N, %)** |  | **(N, %)** | **(N, %)** |  |
| <29 | 11196 (57) | 1805 (8.4) | ≤0.001*** | 11528 (59) | 1473 (6.6) | ≤0.001*** |
| ≥30 | 5990 (27) | 1514 (7.3) |  | 6424 (30) | 1080 (4.7) |  |
| **Hypertension** |  |  |  |  |  |  |
| Normal BP | 13915 (74) | 2718 (14) | ≤0.001*** | 14822 (79) | 1811 (8.8) | ≤0.001*** |
| High BP | 2305 (9.7) | 528 (2.4) |  | 2427 (10) | 406 (0.6) |  |
| **LLD** |  |  |  |  |  |  |
| LLD | 5495 (55) | 1124 (11) | 0.736 | 1012 (6.7) | 111 (0.36) | 0.041* |
| Non LLD | 2423 (24) | 887 (9.5) |  | 11654 (83) | 1643 (10) |  |
| **CVD** |  |  |  |  |  |  |
| CVD | 603 (2.2) | 143 (0.5) | 0.632 | 621 (2.3) | 125 (0.4) | 0.042* |
| Other than CVD | 16788 (81) | 3602 (17) |  | 17835 (86) | 2555 (11) |  |
| **Male** |  |  |  |  |  |  |
| **BMI (kg/m^2^)** |  |  |  |  |  |  |
| <29 | 543 (8.2) | 95 (1.6) | 0.399 | 5627 (61) | 515 (5,4) | ≤0.001*** |
| ≥30 | 4730 (74) | 1007 (17) |  | 2690 (29) | 412 (4.5) |  |
| **Hypertension** |  |  |  |  |  |  |
| Normal BP | 6507(69) | 1173 (18) | ≤0.001*** | 7053 (79) | 753 (7.7) | ≤0.001*** |
| High BP | 1264 (10) | 374 (3.3) |  | 1139 (12) | 184 (1.6) |  |
| **LLD** |  |  |  |  |  |  |
| LLD | 421 (4.9) | 64 (0.5) | 0.449 | 570 (9.3) | 33 (0.65) | 0.065 |
| Non LLD | 6626 (84) | 934 (11) |  | 4836 (81) | 519 (9.1) |  |
| **CVD** |  |  |  |  |  |  |
| CVD | 345 (2.2) | 78 (0.5) | 0.304 | 349 (2.4) | 32 (0.3) | 0.668 |
| Other than CVD | 7888 (76) | 2291 (21) |  | 8520 (88) | 961 (9.1) |  |
| **Female** |  |  |  |  |  |  |
| **BMI (kg/m^2^)** |  |  |  |  |  |  |
| <29 | 5433 (59) | 490 (5.4) | ≤0.001*** | 5347 (58) | 576 (6.2) | 0.004* |
| ≥30 | 3275 (30) | 588 (5.6) |  | 3373 (31) | 488 (4.3) |  |
| **Hypertension** |  |  |  |  |  |  |
| Normal BP | 6993 (80) | 857 (9.5) | 0.201 | 7172 (82) | 676 (7.4) | 0.004* |
| High BP | 968 (9) | 131 (1.3) |  | 966 (9.1) | 133 (1.2) |  |
| **LLD** |  |  | 0.375 |  |  | 0.474 |
| LLD | 384 (4.7) | 59 (0.7) |  | 381 (4.7) | 62 (0.7) |  |
| Non LLD | 6204 (83) | 790 (11) |  | 6160 (84) | 833 (11) |  |
| **CVD** |  |  |  |  |  |  |
| CVD | 244 (2.2) | 28 (0.3) | 0.962 | 244 (2.2) | 28 (0.2) | 0.541 |
| Other than CVD | 8386 (87) | 1053 (11) |  | 8574 (89) | 863 (8.5) |  |

**Table 1c. Association of demographic characteristics with ALT and AST**

Male: ALT (U/L) <40 and ≥40; AST <37 and ≥37

Female: ALT (U/L) <31 and ≥31; AST <31 and ≥31

*Significant at ≤0.05 and ***≤0.001

LLD=Lipid lowering drugs; CVD=Cardiovascular disease; BP=Blood pressure, High (≥140/90 mmHg)
